# Socio-economic factors associated with infant mortality in Italy: an ecological study

**DOI:** 10.1186/1475-9276-11-45

**Published:** 2012-08-16

**Authors:** Laura Dallolio, Valentina Di Gregori, Jacopo Lenzi, Giuseppe Franchino, Simona Calugi, Gianfranco Domenighetti, Maria Pia Fantini

**Affiliations:** 1 Department of Medicine and Public Health, Alma Mater Studiorum – University of Bologna, via San Giacomo 12, 40126, Bologna, Italy; 2 Institute of Public Communication and Education (ICIeF), Institute of Microeconomics and Public Economics (MECOP), University of Lugano, via Buffi 13, 6900, Lugano, Switzerland

**Keywords:** Infant mortality, Income, Income inequality, Unemployment rate

## Abstract

**Introduction:**

One issue that continues to attract the attention of public health researchers is the possible relationship in high-income countries between income, income inequality and infant mortality (IM). The aim of this study was to assess the associations between IM and major socio-economic determinants in Italy.

**Methods:**

Associations between infant mortality rates in the 20 Italian regions (2006–2008) and the Gini index of income inequality, mean household income, percentage of women with at least 8 years of education, and percentage of unemployed aged 15–64 years were assessed using Pearson correlation coefficients. Univariate linear regression and multiple stepwise linear regression analyses were performed to determine the magnitude and direction of the effect of the four socio-economic variables on IM.

**Results:**

The Gini index and the total unemployment rate showed a positive strong correlation with IM (r = 0.70; p < 0.001 and r = 0.84; p < 0.001 respectively), mean household income showed a strong negative correlation (r = −0.78; p < 0.001), while female educational attainment presented a weak negative correlation (r = −0.45; p < 0.05). Using a multiple stepwise linear regression model, only unemployment rate was independently associated with IM (*b* = 0.15, p < 0.001).

**Conclusions:**

In Italy, a high-income country where health care is universally available, variations in IM were strongly associated with relative and absolute income and unemployment rate. These results suggest that in Italy IM is not only related to income distribution, as demonstrated for other developed countries, but also to economic factors such as absolute income and unemployment. In order to reduce IM and the existing inequalities, the challenge for Italian decision makers is to promote economic growth and enhance employment levels.

## Introduction

The infant mortality rate (IMR) is an indicator of child health [1] as well as of population health. Socio-economic factors affecting the health of the population (such as economic development, general living conditions and social wellbeing) have an impact on the IMR [2,3]. According to the analytical framework proposed by Mosley and Chen, infant or child deaths are seen as attributable to a range of hierarchical determinants that may be proximal (e.g., maternal factors, nutrition deficiency, infections, injuries, health services utilization), intermediate (e.g., access to food, safe water, health services, vaccinations), or distal (e.g., education, unemployment, national income, income distribution, public health spending) [4].

One issue that continues to attract the attention of public health researchers is the possible relationship in high-income countries between income, income inequality and infant mortality (IM) [5,6]. According to the income inequality hypothesis, referred to as the “big idea”, once a society progresses beyond the point of absolute deprivation, then it is the distribution of income within the society that affects health outcomes [7]. A significant association in wealthy nations between IM and income distribution, but not with absolute income were found in two recent ecological cross-sectional studies of OECD (Organisation for Economic Co-operation and Development) countries [8,9] and in a systematic review [10]. Conversely, Schell conducted a study of 152 high, middle and low income countries, and did not find a significant association between income inequality and IMR in high income countries [11]. Recently, Olson found that both income and income inequality affect IM in the USA [6]. Moreover, Regidor showed that in 21 high-income countries, the relationship between IM and income inequality, demonstrated in 1995, disappeared in 2005 [5].

In summary the relationship between income, income inequality and IMR in high-income countries seems to vary according to specific characteristics of the countries, socio-economic indicators chosen for the analyses and across years. Therefore, it could be beneficial to investigate the association between IM and socio-economic factors in Italy in order to tackle existing health inequalities. To our knowledge, no such research has been undertaken in Italy, a country characterized by one of the lowest IMR in Europe (3.3/1,000 live births in 2008), but with large inter-regional disparities [12], and levels of inequality and poverty that are among the highest measured in wealthy nations [13].

The aim of our study was to assess the associations between IM and major socio-economic determinants in Italy, in an ecological analysis using recent data (2006–2008).

## Methods

The units of observation for the present study were the 20 Italian Regions in the years 2006–2008. We selected, from the existing literature, four potentially important distal socio-economic determinants of IM: income inequality, mean household income, educational attainment and unemployment rate. The Gini coefficient was chosen as a measure of income inequality, and ranged from 0 (total equality) to 1 (total inequality). Data on the Gini coefficient were extracted from the Italian National Institute of Statistics (ISTAT) [14].

As a measure of absolute income we used the mean annual household income. Data on mean household income for 2007 were obtained from the National Institute of Statistics (ISTAT) [15]. These data are derived from a survey carried out yearly by the Italian National Institute of Statistics within a broader project named “Statistics on Income and Living conditions (Eu-Silc)” coordinated by Eurostat. According to the Eurostat definition, the median family income is calculated as the sum of income derived from dependent and independent work, from real and financial capital, from pensions and other public or private funds received by the family, excluding taxes and loans.

The association between IM and both maternal and general education is recognized in several studies [16,17]. We used the percentage of women aged ≥15 educated to first grade secondary level (i.e. at least 8 years of education) as a measure of educational attainment because we realized that most women with only a primary education level (i.e. 5 years of education) are no more in their childbearing age. Data on educational attainment in 2007 were extracted from the Health for All-Italy database [18].

We assessed the association between unemployment and IM because the unemployment rate in an area is seen as the main indicator of economic success or failure [19]. We chose the percentage of unemployed women aged 15–64 years as a measure of unemployment. Data on unemployment rate were derived from ISTAT [18].

Mean regional data for the years 2006–2008 were used for statistical analyses.

Pearson correlation coefficients were calculated to measure the correlation between IMR and the four socio-economic determinants. Univariate linear regression analyses were performed to determine the magnitude and direction of the effect of the socio-economic variables on IMR. We then carried out a multiple stepwise linear regression, with a significance level of entry and removal equal to 0.05. If certain assumptions are met, and this is the case of our data, linear regression is the best method for quantifying the strength of the linear relationship between a dependent variable and one or more predictors. Regression diagnostics did not reveal the presence of influential observations nor evidence of spatial dependence in the residuals.

All analyses were weighted by the mean number of live births for the period 2006–2008, in each region. Weights were calculated as the average of live births on 1^st^ January 2006 and 31^st^ December 2008. Statistical analyses were carried out using STATA software, version 11 (StataCorp LP, College Station, TX, USA).

## Results

Table 1 shows summary statistics of IMR and socio-economic variables. IMR in Italy in 2006–2008 was 3.4/1,000 live births ranging from 1.98 in Friuli-Venezia Giulia to 4.82 in Calabria. The mean Gini coefficient was 26.7% (standard deviation (SD) = 19.95%), with the lowest value in Veneto (Gini coefficient 23.1%) and the highest in Campania (Gini coefficient 30.9%). The mean family income ranged from 25,952 Euro in Sicily to 39,487 Euro in Lombardy, with a mean of 33,452 Euro (SD = 4,547.15 Euro). The proportion of women with at least 8 years of education ranged from 26.67% in Campania to 37.41% in Lazio, with an overall mean of 30.09% (SD = 2.9%). The unemployment rate was lowest in Trentino-South Tyrol (2.79%) and highest in Sicily (13.07%), with a mean across Italy of 6.5% (SD = 3.4%).

**Table 1:** Summary statistics

**Variable**	**N**	**Mean**	**SD**	**Min**	**Max**
Gini coefficient (%)	20	26.70	18.95	23.10	30.90
Mean household income (thousands of Euros)	20	33.45	4.55	25.95	39.49
Unemployment rate for women aged 15–64 (%)	20	6.50	3.40	2.79	13.07
Proportion of women with ≥ 8 years of education (%)	20	30.90	2.90	26.67	37.41
Infant mortality rate ( ‰)	20	3.40	0.70	1.98	4.82

*SD*: Standard deviation.

**Figure 1:**
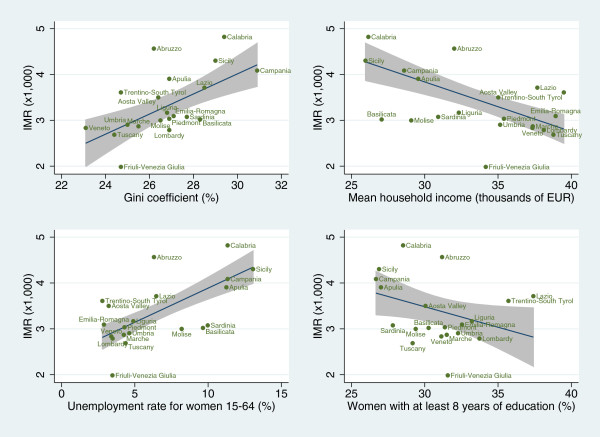
** Linear regression models showing the relationships between income inequality, income, unemployment, education and infant mortality rates in Italian regions.** IMR: infant mortality rate. Note. Grey area: 95% confidence interval (CI) for the linear fit.

The IMR was strongly and positively correlated with the Gini coefficient (r = 0.70; p < 0.001) and the unemployment rate (r = 0.84; p < 0.001). We also found a negative and significant correlation between IMR and mean household income (r = −0.78; p < 0.001) and the proportion of women with at least 8 years of education (r = −0.45; p = 0.046). These results were confirmed by the coefficients of the linear regression analyses that indicate a significant strong relationship between IMR and Gini coefficient, unemployment rate and mean household income (p ≤ 0.001). Last, we found a weaker relationship between IMR and the proportion of women with at least 8 years of education (p = 0.046) (Figure 1 and Table 2).

**Table 2:** Univariate linear regression models on infant mortality rates across Italian regions

	**IMR**		
	**Coef. (95% CI)**	**R**^**2**^	**p-value**
Gini coefficient	0.22 (0.11, 0.33)	0.49	0.001
Mean household income	−0.11 (−0.15, –0.06)	0.60	<0.001
Unemployment rate	0.15 (0.10, 0.20)	0.70	<0.001
Proportion of women with ≥ 8 years of education	−0.09 (−0.18, –0.00)	0.20	0.046

*IMR*: infant mortality rate; *Coef*.: regression coefficient.

Regarding the interpretation of the regression coefficients, our results indicated that for a 1% increase in the Gini index, the model predicted a 0.22 ‰ increase in IMR; for a 1,000 Euro increase in mean household income, the model predicted a 0.11 ‰ decrease in IMR; for a 1% increase in unemployment rate, the model predicted a 0.15 ‰ increase in IMR; for a 1% increase in the proportion of women with at least 8 years of education, the model predicted a 0.09 ‰ decrease in IMR.

In the stepwise regression analysis, only unemployment rate was retained as an independent variable in the model (Table 3).

**Table 3:** Stepwise multiple linear regression model on infant mortality rates across Italian regions

	**Step 1**	
	**Coef. (95% CI)**	**p-value**
Gini coefficient	0.03 (−0.10, 0.16)	0.661
Mean household income	−0.04 (−0.16, 0.09)	0.547
Unemployment rate	0.14 (−0.02, 0.30)	0.075
Proportion of women with ≥ 8 years of education	0.07 (−0.03, 0.16)	0.143
	**Step 2**	
	**Coef. (95% CI)**	**p-value**
Gini coefficient	–	–
Mean household income	−0.04 (−0.15, –0.08)	0.519
Unemployment rate	0.16 (0.02, 0.29)	0.027
Proportion of women with ≥ 8 years of education	0.08 (−0.01, 0.16)	0.068
	**Step 3**	
	**Coef. (95% CI)**	**p-value**
Gini coefficient	–	–
Mean household income	–	–
Unemployment rate	0.19 (0.13, 0.26)	<0.001
Proportion of women with ≥ 8 years of education	0.07 (−0.01, 0.14)	0.076
	**Step 4**	
	**Coef. (95% CI)**	**p-value**
Gini coefficient	–	–
Mean household income	–	–
Unemployment rate	0.15 (0.10, 0.20)	<0.001
Proportion of women with ≥ 8 years of education	–	–

Notes. Step 1: R^2^ = 0.76; F-test (p-value) = 12.01 (<0.001). Step 2: R^2^ = 0.76; F-test (p-value) = 16.78 (<0.001). Step 3: R^2^ = 0.75; F-test (p-value) = 25.81 (<0.001). Step 4: R^2^ = 0.68; F-test (p-value) = 42.05 (<0.001).

## Discussion

Overall our data confirm low national rates of IM in Italy. Despite this good overall result, the IMR shows important variations among geographical areas, generating disparities between Northern and Southern regions. In 2008, the most recent data available, the IMR was 3.3 per 1,000 live births, one of the lowest in Europe, and ranged from 2.9/1,000 in Northern regions to 4.1/1,000 in Southern regions [20].

Our study showed that all four socio-economic determinants were significantly associated with IMR. In detail, we found that both income and income inequality were associated with IM. These results are consistent with those reported by Olson in the USA [6], but only partially consistent with evidence from cross-sectional studies (in countries including Italy) [8,9], which concluded that in developed societies IM is associated with income inequality but not with absolute income. In line with several studies [16,17] our results also indicated a significant inverse association between IM and female education.

We determined that, among the four socio-economic factors, the unemployment rate showed the strongest correlation with IMR, and female education the weakest. In the multiple regression model, the unemployment rate in Italy proved to be the most suitable indicator of absolute deprivation compared with the other socio-economic factors. As shown by Janlert (2009), a number of different models may be used to explain the links between unemployment and ill-health [21]. The economic deprivation model proved to be the most successful. This sociological model implies that unemployed people have less money and a lack of money will worsen the conditions for good health. The model also suggests a potential solution to the problem: by giving the unemployed support for subsistence, the most deleterious effects of unemployment could be alleviated [21]. It is important to point out that the Italian unemployment insurance system remains one of the least generous within the OECD countries [22]. Thus, it could be important to build an effective social safety net and promote policies aimed at increasing employment and income.

Our study was not designed to examine or explain inequalities between the different areas of Italy. However, we speculate that regional differences in socio-economic factors, where Southern regions are more deprived and have a very high unemployment rate, can reflect disparities in IMR [23-25]. Public health services in Italy are already experiencing the impact of the ongoing economic crisis. Ensor et al. (2010) assessed that recessions do have a negative association with maternal and infant health outcomes [26]. According to this perspective, socio-economic factors could impact not only on IMRs, but also explain regional disparities.

The study has several limitations. It is an ecological study and therefore does not provide evidence of causality. Furthermore, the level of geographical aggregation could influence the association between socio-economic factors and IM, and we limited the study to a regional level. We do not know whether the use of data at a more local geographical level (provinces) would have affected the results. Despite these limitations, our research suggests that in Italy, as in the USA, both income and income inequality are associated with IMR, but that the unemployment rate is the major socio-economic determinant.

## Conclusions

This study suggests that, in Italy, income inequality, mean household income and unemployment affect IM. Gains in infant health may be achieved through reductions in income inequalities and, in particular, through improvement in employment levels and economic growth. The challenge is to ensure that the policy responses do not exacerbate the existing inequalities but promote employment, particularly in the poorer Southern regions.

## Competing interests

The authors declare that they have no competing interests.

## Authors’ contributions

Paper conception and drafting of manuscript: LD, VDG. Analysis and interpretation of data: JL, SC. Acquisition of data: GF. Critical revision: GD, MPF. All authors read and approved the final manuscript.

## References

[B1] Gray R Hollowell J Brocklehurst P Graham H Kurinczuk JJ Inequalities in infant mortality project briefing paper 2. Health inequalities infant mortality target: technical background 2009 National Perinatal Epidemiology Unit, Oxford https://www.npeu.ox.ac.uk/infant-mortality

[B2] Reidpath DD Allotey P Infant mortality rate as an indicator of population health J Epidemiol Community Health 2003 57 344 346 10.1136/jech.57.5.344 12700217 PMC1732453

[B3] Kurinczuk JJ Hollowell J Brocklehurst P Gray R Inequalities in infant mortality project briefing paper 1. Infant mortality: overview and context 2009 National Perinatal Epidemiology Unit, Oxford https://www.npeu.ox.ac.uk/infant-mortality

[B4] Mosley WH Chen L An analytical framework for the study of child survival in developing countries Popul Dev Rev 1984 10 Suppl 25 45 PMC2572391 12756980

[B5] Regidor E Martínez D Santos JM Calle ME Ortega P Astasio P New findings do not support the neomaterialist theory of the relation between income inequality and infant mortality Social Science & Medicine 2012 10.1016/j.socscimed.2011.09.041 22200093

[B6] Olson ME Diekema D Elliott BA Renier CM Impact of income and income inequality on infant health outcomes in the United States Pediatrics 2010 126 6 1165 1173 10.1542/peds.2009-3378 21078730

[B7] Wilkinson RG Pickett K The Spirit Level: Why More Equal Societies Almost Always Do Better 2009 Penguin, United Kingdom

[B8] Pickett KE Wilkinson RG Child wellbeing and income inequality in rich societies: ecological cross sectional study BMJ 2007 335 7629 1080 10.1136/bmj.39377.580162.55 18024483 PMC2094139

[B9] Lindström C Lindström M Social capital, GNP per capita, relative income, and health: an ecological study of 23 countries Int J Health Serv 2006 36 4 679 696 10.2190/C2PP-WF4R-X081-W2QN 17175841

[B10] Spencer N The effect of income inequality and macro-level social policy on infant mortality and low birthweight in developed countries-a preliminary systematic review Child: Care, Health & Development 2004 30 6 699 709 10.1111/j.1365-2214.2004.00485.x 15527480

[B11] Schell CO Reilly M Rosling H Peterson S Exstrom AM Socioeconomic determinants of infant mortality: A worldwide study of 152 low-, middle-, and high-income countries Scandinavian Journal of Public Health 2007 35 288 297 10.1080/14034940600979171 17530551

[B12] Fantini MP Stivanello E Dallolio L Loghi M Savoia E Persistent geographical disparities in infant mortality rates in Italy (1999–2001): comparison with France, England, Germany, and Portugal Eur J Public Health 2006 16 4 429 492 10.1093/eurpub/ckl009 16524941

[B13] Brandolini A Income Inequality in Italy: Facts and Measurement http://www.sis-statistica.it/files/pdf/atti/Atti%20pubblicati%20da%20Cleup_55_77.pdf

[B14] The Italian National Institute of Statistics ISTAT datawarehousehttp://dati.istat.it/

[B15] ISTAT: Condizioni di vita e distribuzione del reddito in Italia Anno 2008 Life conditions and income distribution in Italy 2008 http://www.lavoro.gov.it/NR/rdonlyres/B12DA14F-AA6B-4734-823F-3439FF53BF98/0/conddivitaistat2009.pdf

[B16] Arntzen A Mortensen L Schnor O Cnattingius S Gissler M Andersen AM Neonatal and postneonatal mortality by maternal education–a population-based study of trends in the Nordic countries, 1981–2000 Eur J Public Health 2008 18 3 245 251 10.1093/eurpub/ckm125 18160387

[B17] Jiménez-Rubio D The impact of fiscal decentralization on infant mortality rates: evidence from OECD countries Soc Sci Med 2011 73 9 1401 1407 10.1016/j.socscimed.2011.07.029 21920653

[B18] Health for All –Italia ISTAThttp://www.istat.it/it/archivio/14562

[B19] Shaw M Galobardes B Lawlor DA Lynch J Wheeler B Davey Smith G The handbook of inequality and socioeconomic position. Concepts and measures 2007 The Policy Press, Great Britain

[B20] Health for All –Italia ISTAT 2008 http://www.istat.it/it/archivio/14562

[B21] Janlert U Hammarström A Which theory is best? Explanatory models of the relationship between unemployment and health BMC Publ Health 2009 9 235 10.1186/1471-2458-9-235 PMC2720386 19602230

[B22] OECD Employment Outlook 2011 – How does ITALY compare? http://www.oecd.org/dataoecd/8/42/48683274.pdf

[B23] Costa G Marinacci C Caiazzo A Spadea T Individual and contextual determinants of inequalities in health: the Italian case Int J Health Serv 2003 33 4 635 667 10.2190/AM8R-K0DY-F7PM-3RNP 14758854

[B24] Bonati M Campi R What can we do to improve child health in southern Italy? PLoS Med 2005 2 9 e250 10.1371/journal.pmed.0020250 16104833 PMC1188255

[B25] Baccile G Valerio M The 150 years anniversary of Italy: a memorandum from the South Assist Inferm Ric 2011 30 2 110 112 10.1702/845.9398 21747572

[B26] Ensor T Cooper S Davidson L Fitzmaurice A Graham WJ The impact of economic recession on maternal and infant mortality: lessons from history BMC Publ Health 2010 10 727 10.1186/1471-2458-10-727 PMC3002333 21106089

